# Plasma NfL Levels Across Cognitive Stages and the Impact of Comorbidities in a Thai Cohort

**DOI:** 10.1002/alz70856_106886

**Published:** 2026-01-11

**Authors:** Chatchawan Rattanabannakit, Kristsana Khuranae, Natthamon Wongkom, Pathitta Dujada, Paphawadee Phoyoo, Leatchai Wachirutmanggur, Vorapun Senanarong

**Affiliations:** ^1^ Faculty of Medicine Siriraj Hospital, Mahidol University, Bangkok, Thailand

## Abstract

**Background:**

Elevated plasma neurofilament light chain (NfL) is a promising biomarker for dementia diagnosis. However, it has also been reported to be associated with age, body mass index (BMI), and comorbidities such as diabetes, hypertension, chronic kidney disease (CKD), stroke, and cardiovascular diseases. This study aimed to investigate the relationship between plasma NfL levels, cognitive performance, and activities of daily living (ADL) in a Thai population, as well as the comorbidities influencing NfL levels.

**Method:**

A pilot study was conducted with 48 participants categorized into normal cognition (*n* = 4), mild cognitive impairment (MCI)(*n* = 18), and dementia (*n* = 26) groups. Plasma NfL levels were analyzed using Single Molecule Arrays (SiMoA) technology. Statistical analyses, including Mann‐Whitney U Test and Spearman's rho correlations, were used to examine associations between NfL levels and other variables.

**Result:**

The mean age of participants was 70.8±12.0 years, and 58.3% were female. Median plasma NFL levels were significantly higher (*p* <0.05) in the dementia group (44.3, IQR45.9) compared to the MCI (18.7, IQR13.9) and normal cognition groups (8.6, IQR15.8). Higher NFL levels were significantly associated with lower cognitive scores, including Thai Mental State Examination scores (r=‐0.58, *p* <0.001) and Montreal Cognitive Assessment scores (r=‐0.55, *p* <0.001). They also correlated with poorer function, as measured by the sum of basic and instrumental ADL scores, and Clinical Dementia Rating scale‐sum of boxes (r=0.61 and 0.64, respectively, *p* <0.001). Plasma NFL levels were significantly correlated with serum albumin (r=‐0.37, *p* = 0.028) and BMI (*r* =‐0.37, *p* = 0.023). Age and comorbidities, including diabetes, hypertension, dyslipidemia, CKD, cardiovascular and cerebrovascular disease, levels of blood urea nitrogen, creatinine, liver enzymes, lipid profiles, and thyroid functions, did not significantly influence NfL concentrations. However, higher creatinine (r=0.31, *p* = 0.051) and a history of CKD (*p* = 0.054) showed a trend toward an associate with elevated plasma NfL levels.

**Conclusion:**

In our pilot study, plasma NfL levels are significantly associated with dementia stages, ADL, serum albumin, and BMI and showed a trend toward an associated with renal dysfunction. The interplay between systemic health and NfL levels underscores the need for comorbidity assessments in dementia research, laying the foundation for future studies to validate NfL and expand its applicability across diverse populations.

